# New Medical Periodicals

**Published:** 1850-09

**Authors:** 


### ARTICLE III.

### NEW MEDICAL PERIODICALS.

*The New York Medical Gazette and Journal of Health.*—Edited by D. Meredith Reese, M. D., LL. D.

We are in the receipt, regularly, of this new medical weekly, the sixth number of which is before us.

It keeps pace with medical matters as they transpire in and about the great metropolis and its vicinity, and pays especial attention to quackery in its various forms. But designed, as it is, for a general, as well as a professional circulation, we should think it would be hard to stear clear of difficulties, even with the learned Dr. Reese at the helm; for the general reader would very likely think what the profession would regard a palpable hit at the quack, as decidedly in his favor. And the idea of bringing up the popular intelligence to a standard in medical matters that shall enable people generally to see the true doctrine in medicine without giving each man, woman and child a thorough medical education is utopian in the extreme.

We hope the *Gazette* may find no reason to regret its mixed nature, in consequence of difficulties of this kind, and that it will have a long and prosperous career. A weekly medical journal in the city of New York, was a desideratum which we are glad to see supplied.

*Southern Medical Reports.*—Just as this number is going to press, we have received the first number of this work, and also the editor's explanation of the reason why it was not forwarded sooner, for both of which our thanks are due.

The work is printed in the best style, containing 472 octavo pages, and from the ability of the Editor and the importance of the subjects discussed, we have no doubt it is an exceedingly interesting and valuable publication. Our time has not, however, permitted a perusal of it, and we must defer a more extended notice to a future number.

*The New Hampshire Journal of Medicine.*—Edited by Edward H. Parker, A. M., M. D., Aug. 1st, 1850. Concord, N. H.

The first number of a new journal with the above title is on our table. It is proposed to be issued monthly, 32 pages in each number, at the rate of one dollar per annum.

It is very neatly printed, and filled with interesting matter. In the salutatory address of the editor he says:—

"As to our matter, we wish it to be distinctly understood that we do not propose to spare ourself to such an extent as to depend upon extracts from other journals. Our hope is to be furnished by the profession with reports of cases and other articles sufficient to fill our pages. But if we must seek for aid from other sources, it will be, as in our present article on Chlorosis and its treatment, by drawing from books not before made accessible to the profession. In this way we shall not furnish to any one a mere reprint of what he has already seen; for, though we do not expect to rival periodicals like the *London Lancet*, we will not be their copyist."

We wish the enterprise abundant success.

A prospectus has been issued for the publication of a medical journal at Keokuk, Iowa. It is be called the *Western Medico-Chirurgical Journal*, and will be issued in monthly numbers of 24 pages each, at $2 per annum, under the editorial charge of Professors Sanford and Armor.

We have seen notices in our exchanges saying that a new medical journal would be issued in Baltimore soon.

The *Transylvania Medical Journal* is hereafter to be published in Louisville, Ky.

Dr. Geo. Mendenhall has become one of the Editors of the *Western Lancet*. He enjoys a good reputation. E.

